# Are vesicular neurotransmitter transporters potential treatment targets for temporal lobe epilepsy?

**DOI:** 10.3389/fncel.2013.00139

**Published:** 2013-08-30

**Authors:** Joeri Van Liefferinge, Ann Massie, Jeanelle Portelli, Giuseppe Di Giovanni, Ilse Smolders

**Affiliations:** ^1^Center for Neurosciences, Vrije Universiteit BrusselBrussels, Belgium; ^2^Institute for Neuroscience, Ghent University HospitalGhent, Belgium; ^3^Department of Physiology and Biochemistry, University of MaltaMsida, Malta

**Keywords:** vesicular neurotransmitter transporters, temporal lobe epilepsy, epileptogenesis, antiepileptic drugs, *SLC17*, *SLC18*, *SLC32*

## Abstract

The vesicular neurotransmitter transporters (VNTs) are small proteins responsible for packing synaptic vesicles with neurotransmitters thereby determining the amount of neurotransmitter released per vesicle through fusion in both neurons and glial cells. Each transporter subtype was classically seen as a specific neuronal marker of the respective nerve cells containing that particular neurotransmitter or structurally related neurotransmitters. More recently, however, it has become apparent that common neurotransmitters can also act as co-transmitters, adding complexity to neurotransmitter release and suggesting intriguing roles for VNTs therein. We will first describe the current knowledge on vesicular glutamate transporters (VGLUT1/2/3), the vesicular excitatory amino acid transporter (VEAT), the vesicular nucleotide transporter (VNUT), vesicular monoamine transporters (VMAT1/2), the vesicular acetylcholine transporter (VAChT) and the vesicular γ-aminobutyric acid (GABA) transporter (VGAT) in the brain. We will focus on evidence regarding transgenic mice with disruptions in VNTs in different models of seizures and epilepsy. We will also describe the known alterations and reorganizations in the expression levels of these VNTs in rodent models for temporal lobe epilepsy (TLE) and in human tissue resected for epilepsy surgery. Finally, we will discuss perspectives on opportunities and challenges for VNTs as targets for possible future epilepsy therapies.

## Introduction

Epilepsy is one of the most common acquired chronic neurologic disorders, affecting approximately 1% of the human population and displaying an annual incidence of 50,000–100,000 persons (Pitkänen and Sutula, 2002; Pitkänen and Lukasiuk, 2009; Fridley et al., 2012). The disorder has disastrous implications for the quality of life of the patients, concerning independent living, education and employment, mobility and personal relationships. In Europe alone, health costs increased considerably, reaching more than €15.5 billion (Baulac and Pitkanen, 2008). Despite the availability of a large number of antiepileptic drugs (AEDs), clinically proven to suppress or prevent seizures, in 30–40% of patients symptoms cannot be controlled (Baulac and Pitkanen, 2008; Abou-Khalil and Schmidt, 2012).

Epilepsy is characterized by spontaneous, recurrent seizures (SRS), caused by abnormal synchronized, high frequency neuronal discharges (Pitkänen and Sutula, 2002; Baulac and Pitkanen, 2008; O'dell et al., 2012). Especially in patients with temporal lobe epilepsy (TLE), marked by partial complex seizures of temporal-lobe origin, a progressive development of the disorder is observed (Pitkänen and Sutula, 2002; Baulac and Pitkanen, 2008). Generally, TLE is initiated by an initial precipitating injury, such as status epilepticus (SE), head trauma, brain infection, stroke, or febrile seizures. This insult triggers a cascade of devastating neurobiological events and histological and biochemical changes during a latency period of 5–10 years. In this time range the patient remains free from symptoms or complications, though an epileptic state is being established in the brain, called epileptogenesis, leading to the occurrence of SRS and the diagnosis of epilepsy (Pitkänen and Sutula, 2002; Sharma et al., 2007; Pitkänen and Lukasiuk, 2009; O'dell et al., 2012).

**Figure 1:**
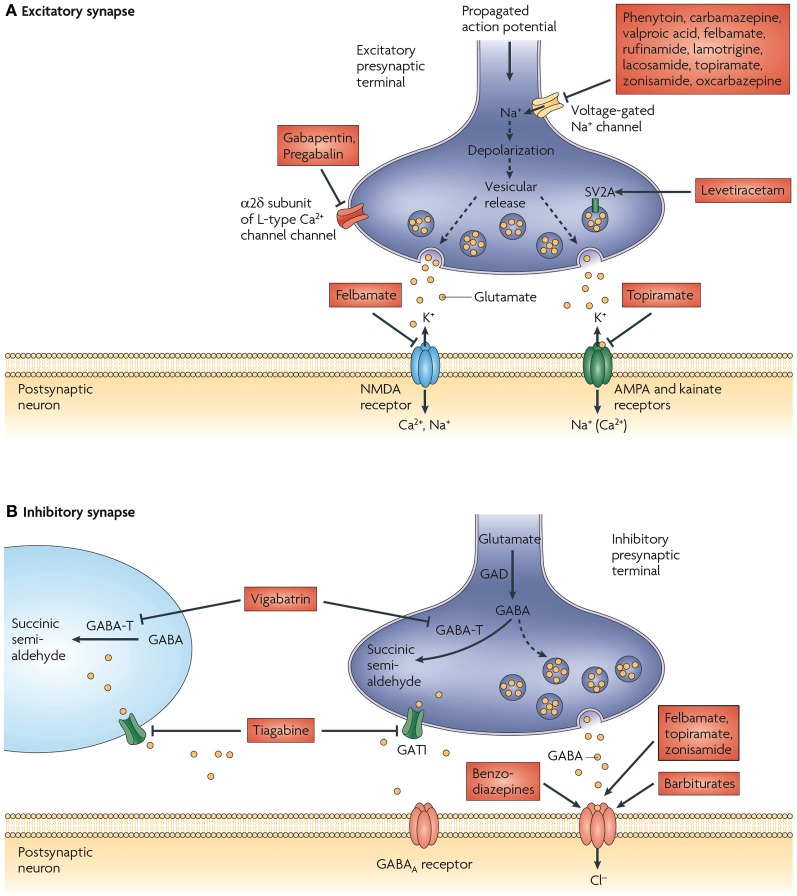
**Proposed mechanisms of action of currently available antiepileptic drugs (AEDs) at excitatory and inhibitory synapses. (A)** Currently available AEDs are thought to target several molecules at the excitatory synapse. These include voltage-gated Na^+^ channels, synaptic vesicle glycoprotein 2A (SV2A), the α 2δ subunit of the voltage-gated Ca^2+^ channel, AMPA (α-amino-3-hydroxy-5-methyl-4-isoxazole propionic acid) receptors, and NMDA (N-methyl-d-aspartate) receptors. Many of the AEDs can modulate voltage-gated Na^+^ channels. This would be expected to decrease depolarization-induced Ca^2+^ influx and vesicular release of neurotransmitters. Levetiracetam is the only available drug that binds to SV2A, which might have a role in neurotransmitter release. Gabapentin and pregabalin bind to the α 2δ subunit of voltage-gated Ca^2+^ channels, which is thought to be associated with a decrease in neurotransmitter release. Excitatory neurotransmission at the postsynaptic membrane can be limited by topiramate (acting on AMPA and kainate receptors) and felbamate (acting on NMDA receptors). **(B)** AED targets at inhibitory synapses have also been proposed. These include the γ-aminobutyric acid (GABA) transporter GAT1 (also known as *SLC6A1*), which is inhibited by tiagabine, leading to a decrease in GABA uptake into presynaptic terminals and surrounding glia, and GABA transaminase (GABA-T), which is irreversibly inhibited by vigabatrin. This decreases the metabolism of GABA in presynaptic terminals and glial cells. The benzodiazepines, barbiturates, topiramate, and felbamate have been found to enhance inhibitory neurotransmission by allosterically modulating GABAA receptor-mediated Cl^−^ currents. However, the action of each of these drugs is different and is dependent on the subunit conformation of the GABAA receptor complex. GAD, glutamic acid decarboxylase. Figure is modified, with permission, from Bialer and White (2010).

The main mechanisms of action of currently available AEDs can be classified into four broad categories: (1) modulation of voltage-dependent sodium, calcium (Ca^2+^) or potassium (K^+^) channels, (2) alterations in γ-aminobutyric acid (GABA)ergic inhibition via actions on GABA_A_ receptors or on GABA synthesis, reuptake or degradation, (3) decreased synaptic excitation via actions on ionotropic glutamate receptors and (4) modulation of neurotransmitter release via presynaptic mechanisms, with most relevant action on glutamate release (Porter et al., 2012) (Figure 1). Despite the fact that some AEDs have proven neuroprotective properties and act by multiple mechanisms of action, they are often only transiently effective and anticonvulsant. Consequently, treatment might be successful at the onset of SRS (silent period), however, as the disease progresses, many patients develop tolerance and become pharmacoresistant (Abou-Khalil and Schmidt, 2012; Kobow et al., 2012; O'dell et al., 2012). Alternative therapies are limited and invasive, including seizure foci resection, vagus nerve stimulation and brain stimulation (Fridley et al., 2012). Obviously, there is an urgent need of non-invasive treatments that prevent or modify the development of epilepsy, in other words, antiepileptogenic or disease-modifying drugs, respectively (Baulac and Pitkanen, 2008). In future validation, new targets should be able to achieve at least one of these two aims (Kobow et al., 2012).

In order to identify new targets, the molecular and cellular mechanisms behind the genesis of epilepsy need to be unveiled. Although countless studies have tried to understand the pathogenesis and progression of TLE, the order of deleterious events during epileptogenesis still remains unknown (O'dell et al., 2012). Moreover, the outcome after the initial insult is considerably variable and at present there are no reliable biomarkers or surrogate disease markers available (Kobow et al., 2012). Current innovative strategies for future AED targets include ion channels and other potential molecular targets (see Meldrum and Rogawski, 2007 and Bialer and White, 2010 for recent reviews) such as neuronal gap junctions (Belousov, 2012; Belousov and Fontes, 2013), neuropeptides (Casillas-Espinosa et al., 2012; Portelli et al., 2012a,b), astrocytic gap junctions and Kir channels (Kovacs et al., 2012; Steinhauser et al., 2012), the cystine/glutamate antiporter or system x_c−_ (De Bundel et al., 2011; Lewerenz et al., 2013), mTOR (McDaniel and Wong, 2011), distinct inflammatory pathways (Vezzani et al., 2013), adenosine kinase (Boison, 2010, 2012), aquaporin channels (Binder et al., 2012; Kovacs et al., 2012) and Ca^2+^-dependent gliotransmission (Carmignoto and Haydon, 2012). It should be taken into consideration that all targets are chosen based on animal models, of which many are not clinically meaningful considering the precipitating cause. In addition, the use of human disease-affected brain tissue is of utmost importance in the search for new drug targets, still there are some disadvantages when human brain tissue is used for investigation. First of all, most patients included in these studies suffered from seizures for many years and secondly, these patients have been treated with specific AEDs. Moreover, it is very unlikely that only one cellular or molecular pathway is responsible for the variety of syndromes and degrees of epilepsy (Kobow et al., 2012).

**Figure 2:**
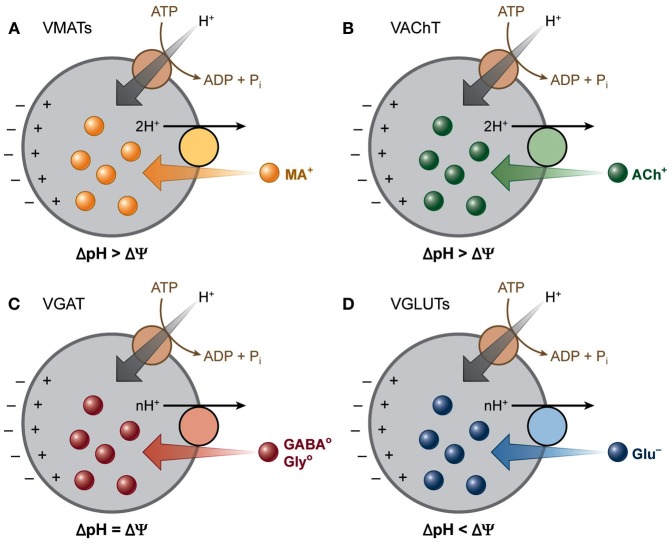
**Vesicular neurotransmitter transporters depend differentially on the two components of the electrochemical gradient of H^+^ (Δμ_H+_)**. A V-ATPase generates a Δμ_H+_ across the vesicle membranes. The vesicular transporters use this gradient to drive the transport of transmitters into secretory vesicles by coupling the translocation of transmitter to H^+^ running down Δμ_H+_. The different vesicular transporters rely to different extents on the two components (Δ pH and Δψ) of this gradient. **(A)** VMATs and **(B)** VAChT transport their positively charged substrates coupled to the exchange of two H^+^, and hence rely primarily on Δ pH. **(C)** GABA and glycine are transported as neutral zwitterions by VGAT, which depends equally on both the chemical and the electrical component of Δμ_H+_. **(D)** VGLUTs transport the negatively charged glutamate and thus rely more on Δψ than Δ pH. [Modified from Chaudhry et al. (2008b) with permission].

An attractive new molecular target for AEDs might be represented by vesicular neurotransmitter transporters (VNTs) (Figure 2). The rationale of their potential use is related on the nature of epilepsy that is characterized by spontaneous, recurrent seizures, caused by abnormal synchronized, high frequency neuronal discharges. Neuronal discharges correspond to chemical transmission, which is essential for normal communication and functioning of the brain. This involves accumulation of neurotransmitters into secretory vesicles, achieved by various types of VNTs, followed by their exocytotic release into the extracellular space (Chaudhry et al., 2008a; Omote et al., 2011). VNTs are small proteins responsible for packing synaptic vesicles with neurotransmitters, thereby determining the amount of neurotransmitter released per vesicle through fusion in both neurons and glial cells. Based on their substrate specificity and amino acid sequence similarity, to date, nine VNTs have been divided into three subclasses; *SLC17*, *SLC18* and *SLC32* gene families. The *SLC17* gene family consists of the three vesicular glutamate transporters (VGLUT1, VGLUT2, and VGLUT3), the vesicular excitatory amino acid transporter (VEAT), and the vesicular nucleotide transporter (VNUT) (Reimer, 2013). The *SLC18* gene family comprises the vesicular monoamine transporters (VMAT1 and VMAT2) for serotonin (5-HT), dopamine (DA), noradrenaline (NE) and histamine and the vesicular acetylcholine transporter (VAChT) (Eiden et al., 2004). Finally, the *SLC32* gene family consists of the vesicular GABA transporter (VGAT) (Gasnier, 2004).

The import of neurotransmitters depends on a proton electrochemical driving force (Δμ_H+_) generated by the vacuolar H^+^-ATPase. Despite the utmost important function of VNTs, the main regulation of this vesicular transport remains unknown. Two mechanisms have been suggested to modulate vesicular transport: (1) influencing the membrane potential (Δψ) by cation and anion fluxes and (2) a direct interaction between the heterotrimeric G-protein, Gα o2 and VNTs (Omote et al., 2011; Blakely and Edwards, 2012; Hnasko and Edwards, 2012) (Figure 2).

Each VNT has always been considered as a specific marker of the respective nerve cells containing that particular neurotransmitter or structurally related neurotransmitters. Recently, it has been observed that several neuronal populations co-release classical neurotransmitters (see for a review Hnasko and Edwards, 2012). The co-transmitters might influence each other's uptake, by influencing the Δμ_H+_, or they can be gathered in distinct vesicles (Hnasko and Edwards, 2012). Glutamate co-release by cholinergic neurons and monoaminergic neurons is most studied and introduced the term of “vesicular synergy,” since vesicular co-accumulation of glutamate by vesicular glutamate transporter 3 (VGLUT3) in cholinergic and serotoninergic neurons, results in higher vesicular import of acetylcholine (ACh) and 5-HT, respectively. The anionic influx of one of the substrates of VGLUT3 (glutamate, Cl^−^ or P_i_) probably creates a lumen-positive Δψ and consequently increases the Δμ_H+_ for ACh and 5-HT vesicular accumulation (El Mestikawy et al., 2011). Due to this co-release neurotransmission might become more complex and expose unraveled roles for VNTs therein. Here we have reviewed the limited literature available on VNTs and epilepsy and their potential role as treatment targets for TLE.

Yet, the only example of a vesicular protein as a target for the treatment of epilepsy is the synaptic vesicle protein 2A (SV2A), the binding site of levetiracetam (LEV). *In vitro* and *ex vivo* binding studies, using SV2A knock-out (^−/−^), heterozygous (^+/−^) and wild-type (^+/+^) mice, identified SV2A as the binding target for LEV. In addition, these transgenic mice were phenotyped in kindling and distinct acute seizure models, unveiling a decreased seizure threshold and accelerated kindling development of the SV2A^+/−^ mice compared to the SV2A^+/+^ mice. SV2A^−/−^ mice, on the other hand, exhibit early severe seizures and die within 2–3 weeks after birth (Kaminski et al., 2012). Other data from these transgenic mice demonstrated the role of SV2A in modulation of vesicular exocytosis (Xu and Bajjalieh, 2001; Budzinski et al., 2009; Chang and Sudhof, 2009; Wan et al., 2010; Yao et al., 2010; Joshi et al., 2013). Although LEV failed in classical seizure screening tests, this drug significantly inhibited the development of seizure kindling and displayed a potential antiepileptogenic effect in the chronic pilocarpine post-SE rat model (Kaminski et al., 2012). A comparative study showed a significant decrease of SV2A protein expression in hippocampus of pharmacoresistant human TLE patients and hippocampus of rats during the latent and chronic phase of the pilocarpine post-SE rat model (van Vliet et al., 2009). These data are consistent with the previously mentioned decreased seizure threshold and accelerated epileptogenesis in SV2A^+/−^ mice (Kaminski et al., 2012). Moreover, these results might explain the loss of the initial efficacy of LEV in refractory TLE patients (Kaminski et al., 2012; Lee et al., 2013). At present, LEV is a worldwide commonly used second generation AED, approved as adjunctive and monotherapy treatment of partial-onset seizures with or without secondary generalization, and adjunctive treatment of myoclonic seizures associated with juvenile myoclonic epilepsy and primary generalized tonic-clonic seizures associated with idiopathic generalized epilepsy (Lyseng-Williamson, 2011). Brivaracetam, a rationally designed LEV derivative, which showed an increased affinity to the LEV-binding site and more potent and complete seizure suppression in animal models of partial and generalized seizures, is currently tested in clinical trial studies (Kaminski et al., 2012).

These data prove that vesicular proteins are accessible and propose a promising role for proteins, involved in vesicle exocytosis, as novel targets for the development of AEDs. Unfortunately, only few studies have followed this concept and brivarecetam is the only new AED in the pipeline targeting a vesicular protein. As outlined for SV2A, results from transgenic mice, distinct acute and chronic rodent epilepsy models and resected human epileptic tissue are crucial to constitute a clear and complete conception of new, potential AED targets. Consequently, in this review we will use the same outline structure to describe the known literature on the different VNTs in epilepsy. Unveiling and understanding the structure of VNTs is of utmost importance to deduce their functional domains and their physicochemical properties in order to develop compounds that can interact with these transporters. Therefore, we will also shortly describe and show the known VNT structure models and the most active and used modulators of their activity. In conclusion, we will point out the remaining challenges for research on VNTs as possible targets for future epilepsy therapies.

## *SLC17* family

The *SLC17* gene family consists of nine members (Reimer, 2013). Three VGLUTs (VGLUT1, VGLUT2, and VGLUT3), the VEAT, and the VNUT are responsible for the vesicular transport of nucleotides and anionic neurotransmitters, glutamate and aspartate. In addition, four sodium-dependent phosphate transporters (NPT1, NPT3, NPT4, and NPT homologue) are part of this family as well. These NPTs are not involved in neurotransmission, but exert voltage driven organic anion elimination of toxic xenobiotics in the kidney and are therefore not further discussed in this review (Omote et al., 2011).

The human SLC17 protein family is categorized as a subgroup of anion transporters within the major facilitator superfamily (MFS). The MFS is the largest group of secondary active transporters, regulating the transport of a wide variety of substrates, such as inorganic ions, sugars, amino acids, and xenobiotics, across cellular and intracellular membranes (Pao et al., 1998; Law et al., 2008). MFS proteins contain 12 transmembrane (TM) helices, divided in two six-TM halves surrounding a central aqueous cavity, in which the substrate-binding site is located. Alternating opening of the binding site on one of both sides of the membrane is achieved by rigid body rotation of the N- and C-terminal halves, called “rocker switch” mechanism (Abramson et al., 2003; Law et al., 2008; Dang et al., 2010). This mechanism enables “uphill” transport of substrate, coupled to “downhill” transport of driving ions such as Na^+^ or protons (Pietrancosta et al., 2012).

### Vesicular glutamate transporters

Glutamate is the most abundant and major excitatory neurotransmitter of the brain, mediating fast synaptic transmission (Casillas-Espinosa et al., 2012; Mehta et al., 2013). Glutamate is crucial for synaptic plasticity (e.g., long-term potentiation, LTP), learning, memory and other cognitive functions. In addition, this neurotransmitter is an important substitute source of energy for neuronal cells in case of glucose deficiency (Mehta et al., 2013). On the other hand, excessive glutamatergic neurotransmission and subsequent glutamate excitotoxicity has been observed in various neurological diseases, such as epilepsy, Alzheimer's disease, Parkinson's disease, multiple sclerosis and stroke. This emphasizes both the crucial and highly toxic role glutamate can play in the brain and the necessity of accurate controlled extracellular levels of glutamate (Casillas-Espinosa et al., 2012; Mehta et al., 2013). VGLUTs are crucial for the storage of glutamate in synaptic vesicles and the subsequent exocytotic release into the synaptic cleft. Synaptic glutamate activates pre- and post-synaptic metabotropic (mGluR) and ionotropic glutamate receptors. The mGluRs are modulators of the synaptic glutamate signal transmission. Activation of group I (mGluR 1, 5) can enhance neuronal excitability, while activation of group II (mGluR 2, 3) and III (mGluR 4, 6, 7, 8) decreases presynaptic glutamate release (see for a review Ure et al., 2006). The ionotropic glutamate receptors include the α-amino-3-hydroxy-5-methyl-4-isoxazolepropionic acid (AMPA), N-methyl-D-aspartate (NMDA) and kainic acid (KA) receptors. These glutamatergic receptors have been intensively studied in both rodent epilepsy models and human epileptic tissue, since excessive activation of these receptors induces glutamate excitotoxicity (see for a review Ghasemi and Schachter, 2011; Matute, 2011; Rogawski, 2013). KA receptors are for example excessively stimulated by local or systemic administered KA, a well-known proconvulsant, used in both acute and chronic rodent epilepsy models to induce convulsions and SE (initial precipitating injury) respectively. Finally, the excitatory amino acid reuptake transporters (EAATs) are of utmost importance for the termination of glutamatergic neurotransmission as well as for the prevention of neurotoxicity. In addition, system x^−^_c_ has been proposed as the most important source of extracellular glutamate in mouse hippocampus and the loss of system x^−^_c_ was shown to decrease the convulsion threshold in distinct acute rodent epilepsy models (De Bundel et al., 2011). Although the involvement of glutamate transporters in epilepsy has been proven in several animal models and human tissue, they are rarely proposed as target for new AED development strategies (Hinoi et al., 2005; De Bundel et al., 2011; Lewerenz et al., 2013).

VGLUTs mediate the import of glutamate into synaptic vesicles. Controlling the activity of these transporters could thus potentially modulate the efficacy of glutamatergic neurotransmission (Takamori, 2006). Indeed, overexpression of VGLUT will increase the amount of glutamate released per vesicle and as such changes in VGLUT expression may affect quantal size and glutamate release under not only physiologic, but also pathologic conditions (Fremeau et al., 2004; Wojcik et al., 2004; Wilson et al., 2005).

**Figure 3:**
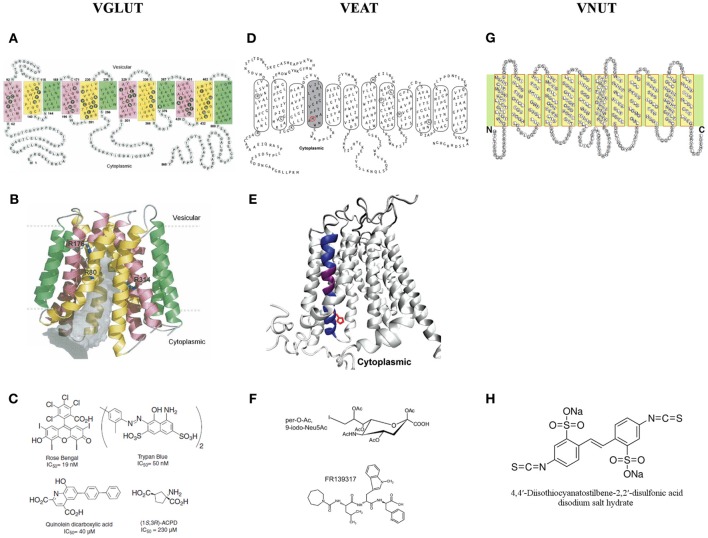
**A two and three dimensional molecular structure of the members of the SLC17 family. (A,B)** The vesicular glutamate transporter 1 (VGLUT1) (Almqvist et al., 2007), **(D,E)** the vesicular excitatory amino acid transporter (VEAT) (Courville et al., 2010) and **(G)** the vesicular nucleotide transporter (VNUT) (Sawada et al., 2008). **(C)** The different families of VGLUT modulators (Pietrancosta et al., 2010). **(F)** The most bioactive analog of sialic acid: per-O-Ac,9-iodo-Neu5Ac and the novel VEAT ligand identified by virtual high-throughput screening: FR139317 (Pietrancosta et al., 2012). **(H)** 4,4′-diisothiocyanatostilbene-2,2′-disulfonate, the only known inhibitor of ATP transport *in vitro*.

In Figures 3A,B, the 2D and 3D molecular structure of VGLUT1 are shown, based on transmembrane segment prediction and topology of bacterial MFS proteins. Filled black circles represent residues facing the center of the pore (gray volume) that is open to the cytoplasmic side. Three arginine residues (R80 from helix 1, R176 from helix 4, and R314 from helix 7) that are exposed to the pore are shown in sticks. The first and last highly variable 60 residues of the N- and C-terminal are not shown in the 3D model (Almqvist et al., 2007).

VGLUT1 and VGLUT2 are complementarily expressed in distinct subsets of glutamatergic neurons in the central nervous system (CNS) (Fremeau et al., 2001; Herzog et al., 2001; Varoqui et al., 2002; Boulland et al., 2004). VGLUT1 is found in astrocytes of the dentate-molecular layers, the stratum radiatum of CA1 hippocampus, the frontal cortex, and the striatum (Bezzi et al., 2004; Zhang et al., 2004; Potokar et al., 2009; Ormel et al., 2012a), while VGLUT2 expression is more restricted and only observed in hippocampal astrocytes (Bezzi et al., 2004). VGLUT3 is expressed in the hippocampus, frontal cortex and in Bergmann glia of the cerebellum. VGLUT3 is relatively more abundant and expressed on distinct synaptic-like microvesicles (SMLVs) than VGLUT1 (Ormel et al., 2012b). Since astrocytes express VGLUTs on SLMVs and since only overexpression of VGLUT3 *in vitro* resulted in increased Ca^2+^ dependent astroglial glutamate exocytosis, it has been postulated that this glutamate transporter is crucial for astroglial glutamate release (Ni and Parpura, 2009; Ormel et al., 2012b). As already mentioned in the introduction, VGLUT3 protein expression is observed in serotoninergic, cholinergic and GABAergic neuronal populations (Fremeau et al., 2002; Gras et al., 2002; Schafer et al., 2002; Gras et al., 2008; Seal et al., 2008).

VGLUT1-immunoreactivity (IR) was reduced in punctate structures of one or several layers of the peritumoral neocortex of patients with epilepsy that was secondary to low-grade tumors, a common cause of epilepsy in which the epileptogenic region presents a loss of neurons and excitatory synapses. This decrease was correlated with gliosis, neuronal loss, and a decrease in the number of asymmetrical synapses (Alonso-Nanclares and De Felipe, 2005). In TLE patients without hippocampal sclerosis, VGLUT1 mRNA levels were decreased, whereas VGLUT1-IR was increased. The authors postulated that this increase could represent a higher vesicular glutamate storage capacity, which may increase glutamatergic transmission, and can contribute to higher extracellular glutamate levels and excitability. In patients with hippocampal sclerosis, on the other hand, both VGLUT1 protein and mRNA levels were decreased in subfields with severe neuron loss, in accordance with the previous study in peritumoral neocortex. Furthermore, upregulated VGLUT1 reactivity could be detected in the dentate gyrus of these patients, indicating that new glutamatergic synapses are formed in the layer with mossy fiber sprouting (van der Hel et al., 2009). As we already mentioned in the introduction, patients are treated with specific AEDs. Consequently, there is a possibility that these drugs influence VGLUT-IR. Indeed, treatment of seizure sensitive Mongolian gerbils with high doses of valproic acid, a Na^+^ channel blocker, but not vigabatrin, a GABA transaminase inhibitor, reduced the VGLUT1/2-IR in the dentate gyrus of these animals. Moreover, the enhanced VGLUT1/2-IR in the seizure sensitive gerbils, compared with the seizure resistant animals, could be closely related to the hyperexcitability of granule cells or the low threshold for seizures in these gerbils (Kang et al., 2005).

Several studies investigated both VGLUT1/2 expression and/or mRNA levels in different animal models of epilepsy. Although models for different types of epilepsy were used, almost all studies showed increased expression of VGLUT1 or VGLUT2 in particular brain regions. Kim et al. showed enhanced VGLUT1-IR in both hippocampi following hypoxic ischemia, although in this model VGLUT2 immunodensity remained unaltered (Kim et al., 2005). On the contrary, VGLUT2 expression was increased in regions of hippocampal heterotopia in methylazoxymethanol exposed animals (Harrington et al., 2007) as well as in the cortex of the genetic absence epilepsy rat from Strasbourg (GAERS) (Touret et al., 2007). In the pilocarpine model of TLE, where a striking loss of mossy cells occurs during the latent period, VGLUT1 mRNA-containing hilar neurons were decreased, together with an associated loss of VGLUT1-containing terminals in the dentate gyrus inner molecular layer. Furthermore, axonal sprouting of granule and pyramidal cells induced aberrant VGLUT1-containing terminals at the chronic stage. VGLUT1-IR was recovered in the inner molecular layer and enhanced in the CA1-CA3 dendritic layers (Boulland et al., 2007). Decreased VGLUT1 levels, detected in the human studies and in the latent period after pilocarpine-seizure induction, are possibly a reflection of the occurring neuronal loss. The enhanced VGLUT1/2-IR in symptomatic animals displaying chronic seizures, on the other hand, may reflect the reorganization of the glutamatergic neurons characterized by sprouting of glutamatergic fibers and associated neosynaptogenesis, together with increased glutamate release in brain regions affected by recurrent seizures (Boulland et al., 2007; Touret et al., 2007).

Therefore, in order to find out whether a severe reduction in VGLUT2 protein can affect seizure generation and thus whether the VGLUT2 protein is crucially involved in the origin of seizure activity, we investigated seizure susceptibility in VGLUT2^+/−^ mice. We showed that, compared to their wild-type littermates, VGLUT2^+/−^ mice are more susceptible to pentylenetetrazole, a chemoconvulsant known to induce generalized seizures (Schallier et al., 2009). These results are suggestive of an important role for VGLUT2 in generalized seizures. However, more studies are needed to fully understand the mechanism behind the altered seizure susceptibility of the VGLUT2^+/−^ mice. It would not be surprising if compensatory up- or downregulation of proteins related to glutamatergic neurotransmission occur. Indeed, although there are no studies that investigated compensations in the VGLUT2^+/−^ mice, increased neuronal synthesis of glutamate, decreased cortical and hippocampal GABA and decreased GLAST (glutamate reuptake transporter) levels have been shown in the VGLUT1^+/−^ mice (Garcia-Garcia et al., 2009).

Recently, mice lacking VGLUT3 were produced to determine the physiological role of this glutamate transporter. These VGLUT3^−/−^ mice exhibit primary, generalized non-convulsive epilepsy (Seal et al., 2008). Although more research should be performed exploring the role of VGLUT3 in seizure generation and epilepsy, this observation strengthens the idea that this transporter is possibly involved in the protection against absence seizures.

Up to date, three types of VGLUT inhibitors (Figure 3C) have been characterized: dyes (e.g., Rose Bengal and Trypan Blue) (Naito and Ueda, 1985; Ozkan and Ueda, 1998; Bole and Ueda, 2005; Almqvist et al., 2007), substituted quinolones (e.g., quinolein dicarboxylic acid) (Bartlett et al., 1998; Carrigan et al., 1999, 2002), and glutamate analogs [e.g., (1S,3R)-ACPD] (Pietrancosta et al., 2010). Of these three types, dyes have the highest affinity toward VGLUTs, especially Rose Bengal, the only competitive inhibitor with the highest affinity of all. The quinolones, on the other hand, display the highest selectivity, while the glutamate analogs have only very low activity on glutamate uptake, which can be explained by the low affinity of VGLUTs toward glutamate itself (Pietrancosta et al., 2010).

### Vesicular excitatory amino acid transporter

The co-localization of glutamate and aspartate was already demonstrated in synaptic vesicles in hippocampal CA1 neurons and SLMVs of pinealocytes (Fleck et al., 1993; Yatsushiro et al., 1997; Gundersen et al., 1998). Moreover, aspartate might be involved in both excitatory and inhibitory synapses in the hippocampus (Gundersen et al., 1991, 1998, 2004). Only recently, Morland and coworkers observed ATP-dependent vesicular uptake of L-aspartate in the brain. Moreover, vesicular L-aspartate uptake, relative to the L-glutamate uptake, was twice as high in the hippocampus as in the whole brain, indicating a crucial role of aspartate signaling in this brain region (Morland et al., 2013). *In vitro* data suggest that NMDA receptor-mediated excitotoxicity is likely caused by extrasynaptic NR1-NR2B NMDA receptor activation by aspartate, which implicates an important role of aspartate signaling in pathologies marked by excitotoxicity. However, the necessity of further studies under physiologic conditions and the development of tools to distinguish aspartate and glutamate postsynaptic responses was emphasized, in order to determine the contribution of aspartate to synaptic transmission (Nadler, 2011). In addition, 30 min after administration of valproate, a broad spectrum AED, aspartate levels are significantly decreased in both excitatory and inhibitory presynaptic nerve terminals in rat hippocampus, whereas glutamate and GABA levels remained unchanged. Consequently, the anticonvulsant effect of valproate might be at least partly achieved by reduced extrasynaptic NMDA receptor-mediated excitatory signaling in the brain (Morland et al., 2012).

Miyaji et al. unveiled the role of sialin as a vesicular aspartate transporter in hippocampal synaptic vesicles and pineal SLMVs (Miyaji et al., 2008). Through *in vitro* reconstitution of mouse and human sialin in liposomes, it was shown that this transporter, which is predominantly expressed in hippocampal neurons (Aula et al., 2004; Yarovaya et al., 2005), is responsible for membrane potential (Δψ)-driven aspartate and glutamate transport into synaptic vesicles in addition to H^+^/sialic acid co-transport in lysosomes. Since aspartate and glutamate are excitatory amino acids, the sialin transporter should be called the VEAT (Miyaji et al., 2008, 2010, 2011).

Mutations in the *SLC17A5* gene, encoding sialin, can elicit two autosomal recessive lysosomal storage disorders: Salla disease and infantile sialic acid storage disease (ISSD). Both disorders are marked by accumulation of sialic acid in lysosomes (Verheijen et al., 1999). Several studies implied a direct correlation between the activity of sialin transport and the severity of the disease phenotype. Mutant forms of sialins in persons suffering from ISSD show complete absence of H^+^/sialic co-transport activity, whereas the mutant forms of sialins found in Salla disease patients still exhibit 20–60% of normal H^+^/sialic co-transport (Morin et al., 2004; Wreden et al., 2005; Myall et al., 2007; Ruivo et al., 2008). Salla disease and ISSD disorders predominantly affect the CNS, eliciting varying degrees of developmental delay in motor and cognitive skills, epilepsy, and premature death and are marked by cytoplasmatic vacuoles and hypomyelination (Prolo et al., 2009).

Incorporation of forms of mouse and human *SLC17A5* protein, associated with Salla disease, in proteoliposomes completely abolished the aspartate and glutamate import, whereas H^+^/sialic acid co-transport was significantly decreased. These results suggest that loss of aspartatergic (and combined glutamatergic) neurotransmission could contribute to the severe neurological defects of Salla disease (Miyaji et al., 2008, 2010, 2011).

Figure 3D represents a 2D topology model for rat and human sialin with manual adjustments. TM4 (shaded) traces out a large aqueous cavity that forms a part of the substrate permeation pathway and displays substrate-induced alterations in accessibility of substituted cysteine residues in TM4. Mutated residues in the lysosomal free sialic acid storage disorders are circled. The 3D model of sialin (Figure 3E), with TM4 colored in blue and purple, is based on the crystal structure of the glycerol 3-phosphate transporter. His-183, a TM4 residue affected by a disease-associated mutation (H183R), is depicted in red in (circle in panel D; side chain in panel E) (Courville et al., 2010).

To further investigate potential mechanisms underlying the pathology of the free sialic acid storage disorders, Prolo et al. characterized sialin-deficient mice. These sialin^−/−^ mice display poor coordination, seizures, failure to thrive, and premature death. Histological characterization of these knock-out mice also revealed prominent vacuolar lesions and a marked decrease in myelin throughout the CNS with the exception of the parasympathetic nervous system. In conclusion, these mice can be used as an appropriate model for free sialic acid storage disorders, since their phenotype appears to be consistent with these disorders (Prolo et al., 2009).

Surprisingly, Morland and coworkers could not observe a difference in ATP-dependent L-aspartate uptake in synaptic vesicles from sialin^−/−^ and sialin^+/+^ mice. Moreover, overexpression of sialin in PC12 cells did not result in significant vesicular uptake of L-aspartate and depolarization-induced depletion of L-aspartate from hippocampal nerve terminals was similar in hippocampal slices from both phenotypes. These *in vitro* results suggest that either sialin is present in insufficient amounts in the vesicular membrane, or sialin does not transport L-aspartate into synaptic vesicles under physiological conditions, contrary to the findings of Miyaji et al. (2008). This discrepancy might be explained by the fact that Miyaji and coworkers used hippocampal P2 fraction for their studies, which is likely to contain non-vesicular membranes and is called the crude synaptosomal fraction. Moreover, a proteomic study (Takamori et al., 2006) did not detect sialin among the other synaptic vesicle proteins, purified from rat brain. On the other hand, no evidence was found of non-vesicular release of L-aspartate, which confirms the exocytotic release in hippocampus, though, after vesicular accumulation by another transporter than sialin (Morland et al., 2013).

Recently, using structure-activity, homology modeling, molecular docking, and mutagenesis studies, the substrate-binding site of sialin has been successfully predicted and a 3D cytosol-open model of sialin has been built and validated. This model predicts small molecule binding to sialin and allows screening of new potential ligands targeting the VEAT. A first pilot virtual high-throughput screening appointed pseudopeptide FR139317 (Figure 3F) to be a competitive inhibitor, showing >100-fold and ~5-fold higher affinity, than the natural substrate, N-acetylneuraminic acid and the most bioactive analog, per-O-Ac,9-iodo-Neu5Ac (Figure 3F) respectively. This increased activity of FR139317 may be due to novel polar interactions, in particular with TM VIII (Tyr-335) (for more details see Pietrancosta et al., 2012).

### Vesicular nucleotide transporter

Burnstock was the first to indicate ATP as a neurotransmitter and eventually its release and co-release was demonstrated in both peripheral and CNS (Burnstock et al., 1972; Burnstock, 1976, 2007). ATP is highly concentrated (up to 100 mM) in neuronal, synaptic vesicles together with other nucleotides, though at lower concentrations, and in granules in adrenal chromaffin cells (Burnstock, 2007). On the other hand, Ca^2+^-triggered ATP release by astrocytes has been repeatedly observed *in vitro* and could be blocked by inhibition of the vacuolar H^+^-ATPase (Newman, 2001; Coco et al., 2003; Pascual et al., 2005; Pangrsic et al., 2007; Zhang et al., 2007; Pryazhnikov and Khiroug, 2008).

Vesicular astrocytic ATP is postulated to be the major, if not the sole, determinant of astrocytic Ca^2+^ wave propagation in hippocampal astrocyte cultures, mediated by G-protein coupled P2Y1 receptors, although further studies should elucidate if astrocytic ATP contributes to Ca^2+^ waves *in vivo* and is involved in neuron-glia interactions (Bowser and Khakh, 2007). In addition, the involvement of another ATP receptor, P2X7, located on neurons and glia, has been observed in the pathophysiology of epilepsy in distinct chronic rodent epilepsy models. However, due to paradoxical results of P2X7 ligands in the pilocarpine and kainate models, additional experiments in distinct rodent epilepsy models, are needed to unveil the role of P2X7 in TLE (Engel et al., 2012).

Astrocytic release of ATP is proposed to play a role in hippocampal heterosynaptic depression. Extracellularly ATP is degraded into adenosine by EctoATPases. Adenosine in turn activates presynaptic adenosine A1 receptors and suppresses glutamate release from other afferents (Pascual et al., 2005; Serrano et al., 2006). Moreover, adenosine has been demonstrated to be an endogenous anticonvulsant and neuroprotectant. Extracellular adenosine levels are controlled by metabolic reuptake through nucleoside transporters and phosphorylation by adenosine kinase (ADK). ADK overexpression and adenosine deficiency have been observed in different rodent models as well as in human tissue resected from patients with hippocampal sclerosis and TLE (Boison, 2012; Masino et al., 2012). Recently Masino et al. have demonstrated that anticonvulsant effects of the ketogenic diet are due to reduction of the expression of ADK in mice and the enhancement of A1 receptor signaling (Masino et al., 2012).

On the other hand it has been shown that adenosine can potentiate hippocampal neuronal activity via binding to adenosine A2a receptors, without affecting presynaptic glutamate release or postsynaptic glutamatergic conductance. Adenosine deficiency in epilepsy can lead to decreased A2a receptor signaling and might be an explanation for comorbidities such as disturbed psychomotor control, sleep disorder and depression (Boison, 2012). Moreover, microdialysis experiments suggest a crucial role of post-synaptic A2a receptors in the anticonvulsant effect of 2-chloroadenosine and the attenuation of evoked glutamate release by 2-chloro-N^6^-cyclopentyladenosine, both well-known adenosine A1 receptor agonists, in the acute local pilocarpine rat model for limbic seizures (Khan et al., 2000, 2001).

VNUT is encoded by the human and mouse *SLC17A9* gene (Reimer and Edwards, 2004; Fredriksson et al., 2008; Sreedharan et al., 2010). Proteoliposomes reconstituted with purified, recombinant *SLC17A9* transporter exhibit Δψ-driven, Cl^−^ dependent ATP transport, similar to the ATP transporter endogenously expressed in synaptic vesicles and chromaffin granules. Suppression of endogenous *SLC17A9* expression in PC12 cells using small interfering RNA (siRNA) decreased KCl-triggered release of ATP, confirming the involvement of this transporter in vesicular storage and subsequent exocytosis of ATP (Sawada et al., 2008).

VNUT is 430 amino acid residues long with 12 putative TM helices with ~23–29% identity and 41–48% similarity to that of other SLC17 members (Figure 3G) (Sawada et al., 2008). To the best of our knowledge we are unaware of the excistence of a VNUT 3D model.

VNUT is predominantly expressed in the brain and adrenal gland. Immunohistochemical studies revealed *SLC17A9* protein expression in astrocytes (Sawada et al., 2008). Whether astrocytic ATP is released by lysosomes (Zhang et al., 2007) or smaller vesicles ~300 nm (Coco et al., 2003; Pangrsic et al., 2007; Pryazhnikov and Khiroug, 2008) is still subject for discussion.

By contrast, ATP release from central neurons is not well-studied. It has only recently been demonstrated that ATP release from cultured rat hippocampal neurons is attenuated by RNAi-mediated knockdown of VNUT. Strong VNUT-IR is observed in the cerebellar cortex and the olfactory bulb. In the hippocampus VNUT has been observed in both excitatory and inhibitory presynaptic neurons (Larsson et al., 2012).

Nowadays, 4,4′-diisothiocyanatostilbene-2,2′-disulfonate (Figure 3H), also known to inhibit VGLUT, is the only inhibitor proven to block ATP transport *in vitro* (Thompson et al., 2005; Sawada et al., 2008).

## *SLC18* family

*SLC18* transporters transport cationic neurotransmitters, such as ACh, NE, 5-HT, DA, and histamine, into synaptic vesicles. The *SLC18* gene family consists of three members: the VMAT1, the VMAT2, and the VAChT (Omote et al., 2011). The three members of the SLC18 family display significant sequence homology (Parsons, 2000; Bravo and Parsons, 2002). Similar to the SCL17 family these transporters consists of 12 TM domains (Wimalasena, 2010).

### Vesicular monoamine transporters

Several studies investigated the role of monoaminergic neurotransmission in the pathophysiology of epileptogenesis and epilepsy and its co-morbidities: anxiety and depression. Direct enhancement of hippocampal extracellular DA or 5-HT levels has been shown to exert both anticonvulsant and antidepressant activities (Smolders et al., 2008), although SRS in pilocarpine-induced epileptic rats are associated with increased mesolimbic dopaminergic activity (Cifelli and Grace, 2012). Rocha et al. observed alterations of the dopaminergic system in the neocortex of patients with TLE (Rocha et al., 2012). Moreover, reduction of brain 5-HT levels facilitates the induction of SE by pilocarpine administration and increases the frequency of SRS. Activation of postsynaptic 5-HT_1A_ receptors shows antiepileptic activity in different TLE models, such as maximal dentate activation (Orban et al., 2013). Furthermore, depletion of 5-HT levels, after administration of 5,7-dihydroxytryptamine into the median raphe nucleus of rats, significantly increased the incidence of pilocarpine-induced SE and the frequency of seizures during the chronic phase of this epilepsy model (Trindade-Filho et al., 2008). NE has been proposed as a potential biomarker for the efficacy of vagus nerve stimulation, an effective adjunctive treatment for medically refractory epilepsy. Vagus nerve stimulation enhances extracellular hippocampal NE levels, which could be at least partly responsible for its seizure-suppressing effect in the intrahippocampal pilocarpine rat model (Raedt et al., 2011). Monoaminergic neurons and their projection fibers are not only found in the cortex, striatum and thalamus, but also in the hippocampus (Joels and Baram, 2009; Sukumar et al., 2012). In addition, monoaminergic control of neurogenesis in the adult midbrain (salamander) and hippocampus (rodents) have been suggested (Park and Enikolopov, 2010; Berg et al., 2011). In conclusion, most studies imply that upregulation of the monoaminergic system could have anticonvulsant activity.

VMAT is responsible for the transport of monoamine neurotransmitters: DA, 5-HT, NE, epinephrine, and histamine, from the cytoplasm into synaptic vesicles via a electrochemical gradient generated by vacuolar type H^+^-ATPase (Schuldiner et al., 1995). Two VMAT isoforms, VMAT1 and VMAT2, have been molecularly cloned (Erickson et al., 1992; Liu et al., 1992). Human VMAT1 is mainly expressed in peripheral endocrine cell populations, such as the adrenal gland, whereas the expression of human VMAT2 is largely confined to neuronal, histaminergic cells. VMAT2 has a consistently higher affinity for most monoamines, particularly histamine (Erickson et al., 1996).

**Figure 4:**
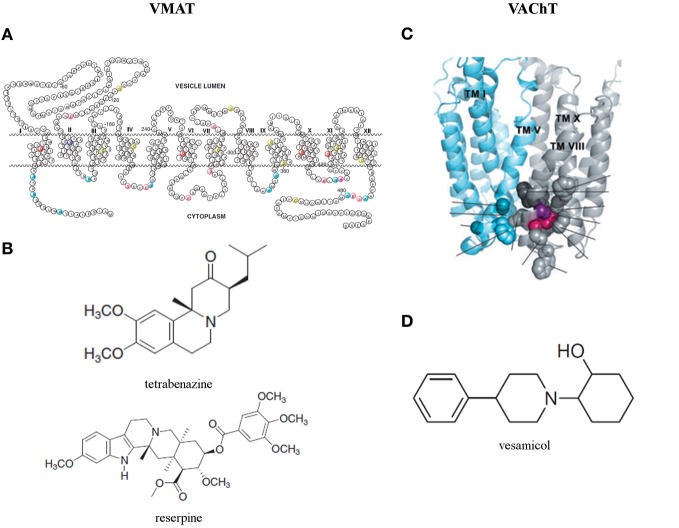
**(A)** Predicted secondary structure of the human vesicular monoamine transporter 2 (VMAT2) and **(B)** two classical and now commonly used VMAT inhibitors: tetrabenazine and reserpine (Wimalasena, 2010). **(C)** Three dimensional homology model of the vesicular acetylcholine transporter (VAChT) (Khare et al., 2010) and **(D)** its most studied inhibitor: vesamicol (Kozaka et al., 2012).

Sequence analysis of human VMAT2 unveilled that most variable regions are located near the N- and C-terminal and in the large glycosylated loop between TMD I and II (Figure 4A) (Wimalasena, 2010). Biochemical studies (Thiriot and Ruoho, 2001; Thiriot et al., 2002) demonstrated that Cys 430 in TMD XI is essential for the recognition and binding of VMAT inhibitors. Moreover, using a recombinant VMAT2 construct with a thrombin cleavage site between TMD VI and VII demonstrated that Cys 117 in the loop between TMD I and II and Cys 324 in the loop between TMD VII and VIII form a disulfide bond in human VMAT2, which contributes to the structural integrity and efficient monoamine transport (Wimalasena, 2010). The structure is predicted by usingTMbase-A database of membrane spanning protein segments. Distinct conserved amino acids in human VMAT1, human VMAT2, rat VMAT1, rat VMAT2, and bovine VMAT2 are colored. The shown amino acid numbering is based on the sequence of human VMAT2 (Wimalasena, 2010).

Three independent groups almost simultaneously published the generation of VMAT2^−/−^ mice. These knock-out mice show lack of feeding behavior and die shortly after birth, which can be partially rescued by amphetamine administration. No differences in monoaminergic cell populations and their projections were observed in VMAT2^−/−^ mice, however, monoamine storage and vesicular release are severely disrupted and brain monoamine levels are dramatically reduced, compared to their wild-type littermates (Fon et al., 1997; Takahashi et al., 1997; Wang et al., 1997). This critical role of VMAT2 in monoamine storage in the CNS and their subsequent exocytotic release is confirmed by *in vitro* and *in vivo* data from VMAT2 heterozygous mice (Fon et al., 1997) and *in vitro* overexpression of VMAT2 in ventral midbrain neurons (Pothos et al., 2000).

A first minor link between epilepsy and VMAT2 was observed in the perinatal asphyxia rat model. Perinatal asphyxia is suggested to induce a variety of brain disorders, including spasticity, epilepsy, mental retardation, attention deficit disorders, and minimal brain disorder syndromes, and could be the initial trigger for subsequent psychiatric and neurodegenerative diseases. Three months after asphyxia was induced in rat pups, tyrosine hydroxylase (TH)-IR was decreased in the striatum, hippocampus, thalamus, frontal cortex, and cerebellum, while VMAT-IR was increased in the striatum. This significant increase of VMAT2 is suggested to be a compensation for deficient dopaminergic/noradrenergic innervation, as a consequence of decreased TH (Kohlhauser et al., 1999).

Following KA-induced SE both TH and norepinephrine transporter (NET), but not VMAT2 mRNA levels are transiently elevated in locus coeruleus (LC) neurons. Noradrenergic LC efferents innervate most forebrain areas and are activated postictally in epilepsy kindling models. Moreover, LC stimulation has been demonstrated to exert seizure-suppressing effects in several epilepsy animal models. Consequently, it is suggested that the increase of TH and NET, responsible for synthesis and reuptake of NE, respectively, could be an adaptive mechanism to restore intracellular NE levels and enable LC neurons to counteract hyperexcitability (Bengzon et al., 1999).

Only recently, it has been shown that VMAT2 protein is mainly expressed in the cytoplasm and axons of neurons of the hippocampus and temporal lobe cortex of rats and humans. Moreover, the expression level of VMAT2 mRNA and protein has been determined in resected neocortices of TLE patients (Jiang et al., 2013) and in the hippocampus and adjacent cortices of rats in different stages of the post-SE pilocarpine model (Jiang et al., 2013). VMAT2 is transiently increased in the acute stage after pilocarpine-induced epileptic seizures, but its expression is clearly decreased after SRS in the hippocampus and temporal lobe cortices of TLE rats. In line with this last observation, the expression level of VMAT2 mRNA and protein in TLE patients is significantly reduced compared to non-epileptic control subjects. Taking previously mentioned studies into account, it is suggested that the transiently increased VMAT2 might be a compensatory response to enhance monoaminergic neurotransmission to counteract hyperexcitability and epileptic seizures. The decrease in VMAT2 expression after SRS is consistent with the known involvement of monoaminergic alterations in epileptic co-morbidities, such as anxiety and depression, and might contribute to the epilepsy disease progression (Jiang et al., 2013).

The potency of two classical and now commonly used VMAT inhibitors, tetrabenazine for VMAT2 and resperine for both isoforms, was confirmed on both human VMAT isoforms for almost two decades (Erickson et al., 1996). Comparison with other VMAT inhibitors point to the optimal positioning of the nitrogen and the carbonyl oxygen of tetrabenazine in the binding site to explain the high affinity of this specific VMAT2 inhibitor. Consequently, since resperine does not contain carbonyl oxygen and nitrogens, its extended hydrophobic tail must provide significant non-specific contribution toward its high binding affinity. Indeed, the binding site of the transporter is known to be particularly hydrophobic. Other inhibitors lacking these important constituents are substantially weaker inhibitors for VMAT in comparison with tetrabenazine and resperine (Figure 4B) (Wimalasena, 2010).

### Vesicular acetylcholine transporter

ACh is secreted both centrally and peripherally, and is known to regulate a plethora of physiological functions. Preclinical and clinical data indicate that cholinergic activity in the entorhinal cortex (EC), which is highly innervated by cholinergic terminals from the basal forebrain (Lewis et al., 1967; Lysakowski et al., 1989; Gaykema et al., 1990), may endorse and maintain epileptiform discharges and kindling (Turski et al., 1989; Saucier and Cain, 1996; Gloveli et al., 1999). The EC propagates cholinergic activity to the stratum oriens of cornu ammonis 1 (CA1), the dentate hilus and many, if not all, interneurons of the hippocampus. Cholinergic functions are altered in the epileptic temporal lobe, however, the exact role and nature of these changes in the pathogenesis of the disease are still as yet to be understood (Friedman et al., 2007). Cholinergic dysfunction (for review see Friedman et al., 2007) and acetylcholine-esterase (AChE) upregulation (Zimmerman et al., 2008) have been observed in the epileptic temporal lobe, however, the exact role of these changes in the pathogenesis of the disease are only recently demonstrated by Gnatek et al. (2012). AChE mRNA and protein levels are fast and strongly increased after SE in mice hippocampus (Gnatek et al., 2012), probably as an initial attempt to maintain homeostasis by reducing ACh levels and subsequently neuronal excitability (Meshorer et al., 2005). Upregulated AChE mRNA was found in principle and inhibitory interneurons, endothelial cells and activated microglia (Gnatek et al., 2012). These observations suggest a possible role in increased blood-brain barrier (BBB) permeability (van Vliet et al., 2007; Weissberg et al., 2011) and in microglial-mediated innate immune response (Vezzani et al., 2011). The upregulation of AChE after SE loosens the brain's immune and anti-inflammatory response and facilitates epileptogenesis. Indeed, transgenic mice overexpressing AChE displayed a robust increase in IL-1β mRNA levels and accelerated epileptogenesis compared to wild-type FVB/N mice (Gnatek et al., 2012).

ACh acts on two different classes of receptors: muscarinic ACh receptors (mAChRs) and nicotinergic ACh receptors (nAChRs). Pilocarpine, a non-selective mAChR agonist, is a widely used chemoconvulsant in both actue and chronic animal epilepsy models (Meurs et al., 2008; Portelli et al., 2009; Bankstahl et al., 2012; Portelli et al., 2012b). The M1 mAChR is crucial for the initiation of pilocarpine-induced seizures, and subsequent maintenance of these seizures necessitates NMDA receptor activation (Maslanski et al., 1994; Smolders et al., 1997). Moreover, M1 receptor knockout mice were found to be highly resistant to pilocarpine-induced seizures (Hamilton et al., 1997). M1 receptor activation highly affects K^+^ conductances, such as K^+^ current blockade thereby leading to a slow depolarization, blockade of the Ca^2+^-dependent slow K^+^ current that is responsible for the after-hyperpolarization that pursues a burst discharge, and blockade of a transient outward K^+^ that regulates excitability in hippocampal neurons (Millan et al., 1993; Friedman et al., 2007). M1 receptor downregulation has been reported following seizure induction in several models, whilst kindled animals show significantly increased expression of both M1 and M3 receptors 28 days following kindling (Friedman et al., 2007). The preponderance of highly sensitive nAChRs is found presynaptically where they stimulate neurotransmitter release, which accordingly influences synaptic efficacy and plasticity, spike-timing-dependent plasticity, frequency-dependent filtering as well as overall signal-to-noise ratio in the cortex (Miwa et al., 2011). Overactivation of nAChRs is thought to be linked to epilepsy (Picard et al., 2006; Miwa et al., 2011). Autosomal dominant nocturnal frontal lobe epilepsy (ADNFLE) is the first human epilepsy for which a mutation has been described and is caused by mutations in the α_4_ or β_2_ subunits of nAChR (Steinlein and Bertrand, 2010). ACh was found to induce seizure-like events in both control and epileptic hippocampal-entorhinal slices, which were completely blocked by the non-specific muscarinic antagonist atropine, partially blocked by the M1 receptor antagonist pirenzepine, and unaffected by the non-specific nicotinic antagonist mecamylamine (Zimmerman et al., 2008).

VAChT, predominantly found in synaptic vesicles, is responsible for loading ACh from the cytoplasm to synaptic vesicles (Prado et al., 2013). Like VGLUTs, VAChT appears to be a very slow transporter and thus is prone to highly influence ACh release (Varoqui and Erickson, 1996; Hori and Takahashi, 2012). VAChT expression levels are known to be affected in Alzheimer's and Huntington's disease (Efange et al., 1997; Smith et al., 2006; Chen et al., 2011), however, not much is known on changes that may occur in an epileptic brain. A 3D model of VAChT has been designed by Schuldiner et al. based on the crystal structure of the MFS protein glycerol-3-phosphate phosphate antiporter (Figure 4C) (Khare et al., 2010).

Only one study is available where VAChT binding-site density was investigated in the human hippocampal formation from epileptic patients that underwent anterior temporal lobectomy. No reduction but rather a slight trend toward increased VAChT binding sites was observed when compared to autopsy controls, as opposed to a significant reduction in mAChRs. This indicates the relative preservation of the cholinergic projecting terminals that is consistent with an axon sparing lesion as opposed to neuronal cell loss in the process of hippocampal sclerosis (Pennell et al., 1999). An explanation for the slight increase in VAChT binding sites may be due to a relative concentration of septohippocampal presynaptic terminals due to synaptic reorganization in the setting of hippocampal atrophy. However, the authors do report an overall reduction in total VAChT per hippocampal formation when compared to autopsy controls due to significant hippocampal atrophy.

In the preclinical setting, there is currently only one study where the role of VAChT in epileptic mechanisms was directly investigated using the pilocarpine animal model of epilepsy (Guidine et al., 2008). Homozygous VAChT knock-down mice were used, resulting in 70% less VAChT expression and a similar deficit in ACh release, since complete VAChT knock-out mice do not survive as a result of compromised respiratory activity (De Castro et al., 2009). These VAChT homozygous knock-down mice were found to show hyperactivity and deficits in spatial memory acquisition as well as lack of behavioral flexibility (Martyn et al., 2012). The authors hypothesized that innate cholinergic hypofunction would lead to receptor upregulation, and hence an increased susceptibility to the convulsive effects of pilocarpine. Their hypothesis held up since the VAChT-deficient mice showed a reduced seizure threshold following pilocarpine administration, which they propose is due to M1 receptor upregulation or overactivation.

Finally, AChE mRNA is increased and mRNA levels of the ACh-synthesizing enzyme choline acetyltransferase (ChAT) and VAChT are decreased following acute stress (Kaufer et al., 1998). Stress is still the most frequently self-reported trigger of seizures. Moreover, enhanced severity of this stress or anxiety increases the risk of subsequent seizures (for review see Friedman et al., 2011). Acute stress transiently enhances acetylcholine transmission and neuronal excitability. Consequently, mRNA levels of the early immediate transcription factor c-Fos, a marker of neuronal hyperexcitation, are robustly elevated. c-Fos has binding sites in the promotors of the previously mentioned cholinergic key genes (AChE, ChAT and VAChT) and will consequently cause long-lasting changes in their expression in neocortex and hippocampus, resulting in a reduction in ACh levels. This delayed secondary phase of suppressed neuronal excitability correlates with the delayed neuropsychiatric pathologies that characterize post traumatic stress disorder, including depression, irritability, and impaired cognitive performance (Kaufer et al., 1998).

The location of the binding sites of Ach and vesamicol, a well-studied inhibitor (Figure 4D), on VAChT are recently investigated by inducing mutations, in and around W331 (Figure 4C) and nearby the luminal end of the transporter. Hitherto, the existence of a spatial cluster of residues close to vesicular lumen, strongly correlated with the affinitiy for ACh and vesamicol. The cluster consists of invariant W331, highly conserved A334 and invariant F335 in TM VIII and invariant C391 in TM X (Khare et al., 2010). However, the mechanistic model for VAChT (Varoqui and Erickson, 1996) displaying two binding sites for ACh, one close to cytoplasm and the other close to the vesicular lumen with transfer of bound ACh between them during transport, cannot be excluded (Khare et al., 2010). Recently, two vesamicol analogues, o-iodo-trans-decalinvesamicol (OIDV) or o-bromo-trans-decalinvesamicol (OBDV), were synthesized and their affinities to VAChT were assessed by *in vitro* binding assays. Both displayed greater binding affinity to VAChT than vesamicol. Moreover, OIDV was able to penetrate the BBB and might be a VAChT imaging probe with high affinity and selectivity (Kozaka et al., 2012).

## SLC32 family

The *SLC32* gene family consists of only one member, the vesicular GABA transporter (VGAT), responsible for the vesicular accumulation of electrically neutral substrates, GABA, and glycine (McIntire et al., 1997; Gasnier, 2004). Since GABA and glycine are known to be inhibitory neurotransmitters, the VGAT transporter is also called the VIAAT (Sagne et al., 1997).

**Figure 5:**
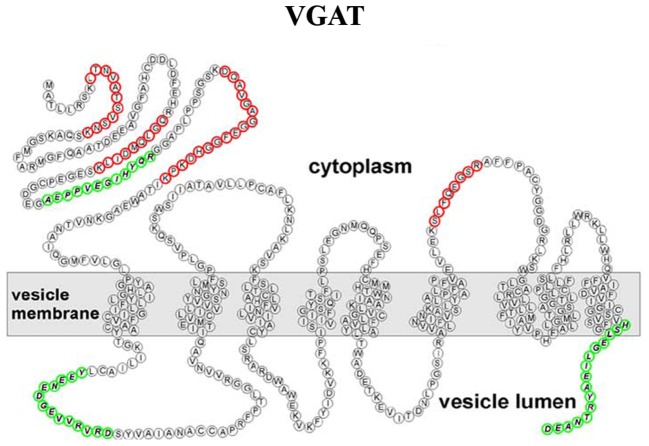
**Refined transmembrane topology of the vesicular GABA transporter (VGAT), the only member of the SLC32 gene family (Martens et al., 2008)**.

Although the VNTs are divided into different families, they were all thought to share a common overall topology with an even number of transmembrane domains and cytosolic localized N- and C-terminals. However, using epitope-specific antibodies and mass spectrometry, Martens and coworkers showed that the VGAT possesses an uneven number of transmembrane domains, with the N-terminus located in the cytoplasm (Figure 5) (Martens et al., 2008).

### Vesicular GABA transporter

As the major inhibitory neurotransmitter in the brain, GABA is an important modulator of hyperexcitability in epilepsy patients and experimental animal models. Some years ago GABA's involvement in epilepsy was suggested (Snodgrass, 1992; Roberts, 2000) and it soon became clear that impairment of GABA functions produced seizures, whereas enhancement resulted in an anticonvulsant effect (Olsen and Avoli, 1997). Accordingly, several old (Holland et al., 1992) and more modern AEDs (Rogawski and Loscher, 2004) act by enhancing the efficacy of GABA-mediated mechanisms proving the GABA theory of epilepsy (Meldrum, 1989; Treiman, 2001). The exception of absence epilepsy proves the role. Indeed, new evidence indicates that increased, rather than decreased, GABAergic inhibition characterizes this generalized form of epilepsy. In particular, enhanced tonic GABA_A_ inhibition, resulting from a malfunction of the astrocytic GABA transporter GAT-1, occurs in thalamocortical neurons of all well-established pharmacologic and genetic models of the disease (Cope et al., 2009; Di Giovanni et al., 2011). Aberrant tonic current has also been shown to have a pathological role in TLE. GABA receptor-mediated inhibition is reduced in the hippocampi of animals during SE (Joshi and Kapur, 2012). SE also causes long-term changes in plasticity of hippocampal GABA receptors consisting of down-regulation of the subunits in the dentate gyrus granule cells, and up-regulation in interneurons. Surprisingly, the level of tonic inhibition in granule cells remains unchanged, consistent with the idea that it is mediated by GABA_A_ receptors containing different subunits (Ferando and Mody, 2012). These findings have important pharmacological implications and extrasynaptic GABA_A_ receptors might become an important AED target.

Of the new AEDs recently launched, tiagabine and vigabatrin (γ-vinyl GABA) act selectively through the GABA-ergic system. The former inhibits neuronal and glial uptake of GABA (including GAT-1) whilst the latter increases the synaptic concentration of GABA by inhibition of GABA-aminotransferase. Other new anticonvulsants are gabapentin and its structure-related pregabalin that are GABA analogues. They easily cross the blood-brain barrier and increase brain synaptic GABA, but their effects are complex and have not been fully characterized (Rogawski and Loscher, 2004; Czapinski et al., 2005). The older benzodiazepine-like AEDs and barbiturates work with different modes of action, through positive allosteric modulation of GABA_A_ receptors. The use of benzodiazepines is currently limited to acute treatment in SE since their use in chronic therapy is characterized by negative side effects, such as sedation, muscle relaxation, and especially the development of tolerance and dependence (Rogawski and Loscher, 2004).

Interestingly, VGAT activity depends equally on both pH and Δψ, differently from the other VNTs (McIntire et al., 1997). *In vitro* studies have shown that VGAT in GABAergic synaptic terminals acts as a co-transporter or symporter for Cl^−^ with GABA (Juge et al., 2009). During refilling, two Cl^−^ ions accompany GABA as it enters the synaptic vesicle. High rates of exchange strongly depend on the size of the pH gradient in the vesicle lumen vs. cytoplasm. A recent study showed that the voltage-gated Cl^−^ ion channel called CLC-3 co-localizes with VGAT in perisomatic synaptic endings on CA1 pyramidal neurons and plays a pivotal role in regulating inhibitory synaptic function by optimizing GABA loading into presynaptic vesicles (Riazanski et al., 2011). CLC-3 might help GABAergic interneurons to maintain rapid firing by ensuring optimal pH differences between the vesicle lumen and cytoplasm, and the availability of protons for rapid GABA loading into vesicles or alternatively act as a Cl^−^ transporter, providing a pathway to clear the vesicle of high Cl^−^ concentrations (Riazanski et al., 2011). These results are noteworthy, particularly that CLC-3^−/−^ mice develop spontaneous seizures that resembled TLE with hippocampal sclerosis and nearly complete bilateral destruction of the hippocampus (Stobrawa et al., 2001). In CLC-3^−/−^ mice, VGAT-dependent GABA release is reduced and feedback inhibition is eliminated leading to limbic seizures. With prolonged seizures, principal neurons become excitotoxic and widespread degeneration occurs (Naegele, 2012).

VGAT plays a fundamental role in embryonic development. Indeed, VGAT^−/−^ mice are not viable and die between embryonic day (E)18.5 and birth. They exhibit a hunched posture and are completely immobile and stiff, most likely symptoms of overexcitation (Wojcik et al., 2006; Saito et al., 2010). VGAT^−/−^ foetuses showed significant increases in both GABA and glycine contents in the forebrain that were not derived from elevated amounts of GABA-synthesizing enzymes. GABA and glycine accumulate in the GABAergic and glycinergic neurons, respectively, but they are not degraded in the glial cells of VGAT^−/−^ mice. These observations bear important consequences for understanding the functional roles of VGAT from the cellular to the whole-body level (Saito et al., 2010). It has been reported that glutamic acid decarboxylase (GAD)65 and not GAD67 forms a complex with heat shock cognate 70, cysteine string protein and VGAT in the nerve terminal. This complex appears to be the necessary machinery required for efficient GABA synthesis and packaging into synaptic vesicles through VGAT (Jin et al., 2003; Buddhala et al., 2009).

VGAT expression increases during development, however, in several brain regions expression levels of VGAT are already high at birth and in some cases they decrease in the course of brain development (Boulland and Chaudhry, 2012). In the hippocampal formation, a shift in the localization of the VGAT nerve terminals has been observed. At early postnatal stages, they are present in dendritic layers while later in the development they target the perikaryon of the principal cells. This redistribution of GABAergic nerve terminals occurs at around P7 which is also the stage at which the postsynaptic response to GABA stimulation switches from membrane depolarization to membrane hyperpolarization. Thus, redistribution of GABAergic nerve terminals together with changes in the expression of Cl^−^ transporters is responsible for the conversion of the GABA action from excitation toward inhibition (Boulland and Chaudhry, 2012). In the normal adult rat hippocampus, VGAT immunoreactive puncta is strongly detected in all pyramidal cell layers of the CA1-3 regions and the granule cell layer of the dentate gyrus (Chaudhry et al., 1998; Kang et al., 2003; Boulland et al., 2007; Choi et al., 2010).

There is only sparse data on VGAT in human epilepsy available. In a case report of Unverricht-Lundborg disease, the most common progressive myoclonic epilepsy, VGAT-IR was decreased in the cortex of the examined patient (Buzzi et al., 2012). In the resected cortex from pediatric epilepsy surgery patients with type II cortical dysplasia GAD-labeled neurons displayed larger somata and VGAT and GABA transporter 1 staining showed a dense plexus surrounding cytomegalic neurons (Andre et al., 2010). Finally, cortical tubers of tuberous sclerosis complex patients showed decreased levels of GAD65, VGAT, GluR subunit 1 and GABA_A_receptor subunits α 1 and α 2 (White et al., 2001). However, until now nothing is known on VGAT in TLE patients.

As far as the experimental models of epilepsy are concerned, the density of VGAT terminals is reduced in an animal model of cortical dysplasia (Zhou and Roper, 2010), in pre-seizure seizure-sensitive gerbil (Kang et al., 2003) and picrotoxin-induced kindling rat hippocampi (Jiang et al., 2004), although VGAT-IR is unaltered in KA-induced seizures (Sperk et al., 2003). Contrasting evidence exists for changes in VGAT in epileptogenesis induced by pilocarpine. One study reported that at 1 day after pilocarpine-induced SE VGAT-IR is not changed (Kwak et al., 2006), instead it is significantly reduced in the dentate gyrus at 1–2 weeks after SE and recovered to the control level at 5 weeks after SE (Kwak et al., 2006). Conversely, other studies support an increased rather than a decreased synthesis of VGAT that occurs after SE and persists until the chronic stage (Boulland et al., 2007; Choi et al., 2010). Indeed, a marked increase in VGAT-IR in all pyramidal cell layers and the granule cell layer of dentate gyrus due to an up-regulation of VGAT mRNA synthesis at 1–2 weeks reaching a maximum level of labeling at 12 weeks after SE has been reported (Boulland et al., 2007; Choi et al., 2010). Therefore, the increased synthesis of VGAT is likely to lead to an increased GABA release from all remaining GABAergic interneurons. Such increased expression of proteins is involved in presynaptic GABAergic transmission and could be associated with increased activity of remaining interneurons (Boulland et al., 2007). This hypothesis is supported by electrophysiological studies showing that, in chronic pilocarpine-treated animals, interneurons are hyperactive (Cossart et al., 2001).

A marked selective decrease in the number of VGAT mRNA-containing neurons in the hilus of the dentate gyrus has been observed (Boulland et al., 2007). This finding is consistent with the cell death of GAD-containing neurons, previously demonstrated in this model (Obenaus et al., 1993). The reduction of VGAT-IR reported after excitotoxic insult might be also explained by VGAT cleavage. Recently, it has been shown that VGAT is cleaved under KA-induced conditions *in vitro* and *in vivo* giving rise to a truncated product that lacks a punctate synaptic distribution. VGAT cleavage was mediated by calpains, and the stability of the cleavage product suggests that the protease plays a modulatory role rather than a destructive effect in excitotoxic neuronal damage (Gomes et al., 2011). VGAT cleavage by calpain leads to the formation of a new and stable truncated VGAT form (tVGAT), which is not targeted to the synapse. This is expected to decrease the release of GABA by exocytosis, thereby changing the equilibrium between excitatory and inhibitory synaptic activity in the early phases after the excitotoxic insult (Gomes et al., 2011). VGATs are not limited to hippocampal GABA interneurons. Indeed, mRNA is also expressed in the dentate gyrus and in mossy fiber synaptosomes and increase after kindling in rats (Lamas et al., 2001). Therefore, granule cells and their mossy fibers, besides being glutamatergic, contain the machinery for the synthesis and vesiculation of GABA. This further supports the notion that local synaptic molecular changes enable mossy fibers to release GABA in response to enhanced excitability as a protective mechanism in response to seizures (Gomez-Lira et al., 2002).

## Concluding remarks

Epilepsy is one of the most common acquired chronic neurologic disorders, with disastrous implications for the quality of life of the patients. Despite the availability of a large number of AEDs, still 30–40% of patients remain pharmacoresistant (Pitkänen and Sutula, 2002; Baulac and Pitkanen, 2008; Pitkänen and Lukasiuk, 2009; Abou-Khalil and Schmidt, 2012; Fridley et al., 2012). Therefore, there is an urgent need of new non-invasive anti-epileptogenic or disease-modifying treatments (Baulac and Pitkanen, 2008). Several innovative targets for future AED are currently intensively investigated. In this review we focused on the role of VNTs in epilepsy and summarized the available evidence in Table 1.

**Table 1:** **Summary of literature regarding transgenic mice with disruptions in vesicular nucleotide transporters (VNTs) in different models of seizures and epilepsy, as well as the known alterations and reorganizations in the expression levels of these VNTs in rodent models for temporal lobe epilepsy (TLE) and in human tissue resected for epilepsy surgery**.

	**Human epileptic tissue**	**mRNA/protein alterations**	**References**	**Animal epilepsy models/transgenic animals**	**Alterations mRNA/protein/seizure threshold**	**References**
VGLUT1	Punctate structures of the peritumoral neocortex	Protein level ↓	Alonso-Nanclares and De Felipe, 2005	Seizure sensitive gerbils (Snodgrass)	Protein level ↑	Kang et al., 2005
	TLE without hippocampal sclerosis	mRNA level ↓	van der Hel et al., 2009	Hypoxic ischemia (both hippocampi)	Protein level ↑	Kim et al., 2005
		Protein level ↑	van der Hel et al., 2009	Post-SE pilocarpine model [latent period, hilar neurons, dentate gyrus (DG)]	mRNA level ↓	Boulland et al., 2007
	TLE with hippocampal sclerosis (subfields with neuronal loss)	mRNA level ↓	van der Hel et al., 2009	Post-SE pilocarpine model (chronic period, CA1-CA3)	Protein level ↑	Boulland et al., 2007
		Protein level ↓	van der Hel et al., 2009			
	TLE with hippocampal sclerosis (DG)	Protein level ↑	van der Hel et al., 2009			
VGLUT2	No studies			Seizure sensitive gerbils (DG)	Protein level ↑	Kang et al., 2005
				Hypoxic ischemia (both hippocampi)	Protein level unaltered	Kim et al., 2005
				Methylazoxymethanol explosion (hippocampus)	Protein level ↑	Harrington et al., 2007
				Symptomatic GAERS (cortex)	Protein level ↑	Touret et al., 2007
				VGLUT2^(+/-)^	Seizure threshold ↓ (pentylenetetrazole)	Schallier et al., 2009
VGLUT3	No studies			VGLUT3^(-/-)^	Spontaneous absence seizures	Seal et al., 2008
VEAT	No studies			Sialin^(-/-)^	Spontaneous seizures	Prolo et al., 2009
VNUT	No studies			No studies		
VMAT2	Medically intractable TLE (neocortices)	mRNA level ↓	Jiang et al., 2013	Perinatal asphyxia rat model (striatum)	Protein level ↑	Kohlhauser et al., 1999
		Protein level ↓	Jiang et al., 2013	Following KA-induced status epilepticus (locus coeruleus)	mRNA level unaltered	Bengzon et al., 1999
				Acute stage post-SE pilocarpine model (hippocampus and cortex)	Protein level ↑	Jiang et al., 2013
				After SRS post-SE pilocarpine model (hippocampus and cortex)	Protein level ↓	Jiang et al., 2013
VAChT	TLE with hippocampal sclerosis	Protein level ↑ trend	Pennell et al., 1999	homozygous VAChT knock-down mice	Seizure threshold ↓ (pilocarpine)	De Castro et al., 2009
VGAT	Unverricht-Lundborg disease (cortex)	Protein level ↓	Buzzi et al., 2012	Cortical dysplasia (CD)	Protein level ↓	Zhou and Roper, 2010
	Pediatric epilepsy type II (CD) (cortex)	Dense plexus surrounding cytomegalic neurons	Andre et al., 2010	Seizure sensitive gerbils	Protein level ↓	Kang et al., 2003
	Tuberous sclerosis complex (cortical tubers)	mRNA level ↑	White et al., 2001	Picrotoxin-induced kindling	Protein level ↓	Jiang et al., 2004
				KA-induced seizures	Protein level unaltered	Sperk et al., 2003
				Post-SE pilocarpine model (hippocampus, 1 day)	Protein level unaltered	Kwak et al., 2006
				Post-SE pilocarpine model (1–2 weeks only DG)	Protein level ↓	Kwak et al., 2006
				Post-SE pilocarpine model (5 weeks only DG)	Protein level unaltered	Kwak et al., 2006
				Post-SE pilocarpine model (hilus of the DG 1–12 weeks)	Number of VGAT mRNA-containing neurons ↓	Boulland et al., 2007
				Post-SE pilocarpine model (hippocampus 1–12 weeks)	mRNA level ↑ (time dependent)	Boulland et al., 2007
				Post-SE pilocarpine model (hippocampus 1–12 weeks)	Protein level ↑ (time dependent)	Boulland et al., 2007

The future perspectives and challenges to fully elucidate the involvement of VNTs and their subsequent possible use as targets for the treatment of TLE are discussed below.

## VGLUTs

Taken together, all the data here reviewed indicate that VGLUTs might be involved in different types of seizures and epilepsy. As expected VGLUTs are the most studied VNTs, although to date, there are no data available on the expression levels of VGLUT2/3 in human epileptic tissue. Furthermore, activity studies need to be performed. Unfortunately, although several competitive and non competitive VGLUT inhibitors have been identified with affinities into the nanomolar range (Thompson et al., 2005; Chaudhry et al., 2008a; Pietrancosta et al., 2010), there is still a lack of specific *in vivo* VGLUT-inhibitors to further investigate and confirm present consistent results.

In addition, it is worthwhile mentioning that a recent study by Juge et al. linked fasting and excitatory neurotransmission through Cl^−^-dependent regulation of VGLUT activity (Juge et al., 2010). The ketogenic diet is often used to control epilepsy (Joshi et al., 2013). Although fasting does not affect the activity of glutamate reuptake transporters (Bough et al., 2007), the vesicular transporters are possibly targeted by the ketone bodies, in particular acetoacetate and β-hydroxybutyrate, produced by this diet. These metabolites can enter the brain, serving as substrates for energy production in neurons. Ketone bodies can compete with Cl^−^, an anion which is absolutely necessary for optimal VGLUT functioning, by shifting the Cl^−^ dependence to a higher concentration. Furthermore, Juge et al. showed that acetoacetate can reversibly suppress glutamate release and seizures evoked by 4-aminopyridine in the brain. Thus, the competitive interaction between Cl^−^ and ketone bodies can turn VGLUT activity off upon binding of the latter, causing a reduction in glutamatergic neurotransmission *in vivo*. The identification of ketone bodies as physiological modulators of VGLUTs advances the search for new approaches in the development of drugs to treat neurological disorders caused by excessive glutamatergic neurotransmission, such as epilepsy (Juge et al., 2010).

## VEAT

The role of sialin in epilepsy and epileptogenesis is far from known. Moreover, the involvement of sialin in the vesicular loading of aspartate has recently been questioned, based on *in vitro* data from sialin^(−/−)^ mice (Morland et al., 2013). Of course, it cannot be excluded that these transgenic mice develop compensatory mechanisms to counteract the loss of sialin. Consequently, there is a need for the development of specific inhibitors of sialin to confirm the role of sialin as VEAT *in vivo*.

Currently commercial antibodies cannot be used for immunolocalization of sialin and from previous immunohistochemical studies (Aula et al., 2004; Yarovaya et al., 2005), using “home-made” anti-sialin antibodies, presence in synaptic vesicles could not be confirmed. Therefore, production of new distinct anti-sialin antibodies, which can be tested for specificity in both Western Blotting and immunohistochemistry in sialin^(−/−)^ vs. wiltype brain tissue, is necessary.

However, since sialin^(−/−)^ mice clearly exhibit seizures, this transporter, whether or not crucial for vesicular import of aspartate, still might be a novel AED target. Subsequently, the currently available tools should be used to further investigate this role. The seizure threshold of sialin^(+/−)^ can be compared to their wildtype littermates in different animal models for epilepsy and moreover, the expression levels of VEAT in human epileptic tissue should be determined.

## VNUT

The lack of tools is a major issue in order to unravel the possible involvement of VNUT in epilepsy and epileptogenesis. Although ADK overexpression and adenosine deficiency are considered to be pathological hallmarks in distinct rodent models and in human tissue resected from TLE patients (Boison et al., 2012), still nothing is known about mRNA or protein expression levels of VNUT in those tissues. Surprisingly, only one home-made antibody against VNUT has been described until now (Larsson et al., 2012). Moreover, the development of specific inhibitors might provide more insights in the role of VNUT in epilepsy as well.

## VMAT

A few studies, including both animal models for epilepsy and human epileptic tissue, indicate the possible involvement of VMAT in acute seizure generation and epileptogenesis.

Recently, Narboux-Neme et al. generated a conditional deletion of VMAT2 in raphe 5-HT neurons by Cre-recombinase expressed under the control of the 5-HT transporter gene (SERT, slc6a4) promoter. These VMAT2^sert−cre^ mice show an almost complete depletion of 5-HT in the brain. Nonetheless, raphe neurons are normally developed and exert normal innervations of target regions. The conditional VMAT2^−/−^ results in a depressive-like phenotype and an anxiety-like response (Narboux-Neme et al., 2011). To further investigate whether VMAT inhibition would indeed negatively affect the course of disease progression in epilepsy, these inhibitors could be administrated at different stages of the chronic post-SE pilocarpine model (Jiang et al., 2013). Similarly, it would be most interesting to subject the conditional VMAT2^−/−^ mice to chronic models of epilepsy.

## VAChT

The precise role VAChT plays in epilepsy still needs to be unraveled. The sparse data from human epileptic tissue and homozygous VAChT knock-down mice, suggest that inhibition of this VNT might lead to protection against seizures. However, cholinergic neurotransmission in the hippocampus is linked to cognitive functions, such as learning and memory. Hippocampal LTP is negatively affected in homozygous VAChT knock-down mice, which is of great importance in learning and memory processes (Martyn et al., 2012; Bliss and Collingridge, 2013). This indicates that a substantial attenuation of VAChT, consequently leading to hippocampal cholinergic deficits, may not be ideal in the clinical situation. Incidentally, cognitive and behavioral impairments are co-morbidities associated with epilepsy (Berg, 2011). Neuropharmacologists aim to find AED treatments that not only suppress seizures and hinder the epileptogenic process, but also prohibit any cognitive dysfunctions in patients with TLE. In an attempt to prevent or reduce worsening of epilepsy-related co-morbidities together with attenuating epileptic seizures, it would be of interest to investigate the actions of VAChT blockers, in animal models of epilepsy to confirm whether similar effects on seizures, epilepsy progression and cognitive functioning will be obtained as in the VAChT knock-down mouse model.

## VGAT

Despite the fact that no drugs acting specifically on VGAT have been identified to date, it has been shown that vigabatrin inhibits VGAT with an affinity similar to GABA (McIntire et al., 1997), an effect that is present only acutely and is lost after chronic treatment with vigabatrin (Engel et al., 2001). This could explain the proconvulsant effect of vigabatrin during the first hours following its administration (Löscher et al., 1989). Selective high-affinity VGAT inhibitors should broadly impair inhibition and would thus be of limited therapeutic interest. Still, they could have provided probes for *in vivo* imaging of inhibitory nerve terminals in the human brain, with potential applications for monitoring the progression or treatment of neurodegenerative diseases characterized by a loss of GABAergic neurons such as epilepsy, Huntington's disease, and brain ischemia. These transporters are particularly interesting as specific markers for GABAergic neurons whose expression levels could reflect the demand for synaptic transmission and their average activity. This could be used in addition to the earlier described intrahippocampal injection of fluorochromated anti-VGAT-C in mice for specific GABAergic synapses labeling *in vivo* (Martens et al., 2008).

Moreover, an interesting strategy for new AEDs targeting VGAT might be related to its expression. For instance, vigabatrin has been shown to be capable of increasing VGAT-IR in the gerbil hippocampus following spontaneous seizures (Kang et al., 2003). Similarly, the opening of ATP-sensitive potassium (KATP) channels by diazoxide prevented seizures and resulted in an up-regulation of VGAT mRNAs and VGAT protein production in hippocampus, and a down-regulation of GAT-1 and GAT-3 gene and protein expressions in picrotoxin-induced kindling in rats (Jiang et al., 2004).

Consequently, epigenetics and microRNA (miRNA) controllers should be considered as hot topic therapeutic interventions to regulate VNTs expression levels.

Epigenetics is defined as information that is heritable during cell division other than the DNA sequence itself, including DNA methylation or histone tail modifications, which can produce lasting alterations in chromatin structure and gene expression (Kobow and Blumcke, 2011). The methylation hypothesis of Kobow and Blümcke postulates that seizures are able to induce epigenetic chromatin modifications and consequently deteriorate epileptogenesis and contribute to structural brain lesions and cognitive dysfunction (Kobow and Blumcke, 2011). Moreover, parallel as in cancer, epigenetic modifications can affect multidrug transporters and induce pharmacoresistance in epilepsy (Kobow et al., 2013). Distinct histone deacetylase and DNA methyltransferase inhibitors are currently under clinical investigation as possible novel epigenetic treatment strategies (Kobow and Blumcke, 2011). More research on epigenetic chromatin modifications will certainly lead to a better understanding of the pathomechanisms involved in epileptogenesis and to novel possible antiepileptogenic compounds or biomarkers (for review see Lubin, 2012; Roopra et al., 2012). For example in prostate cancer, *SCL18A2* hypermethylation was observed in ~90% of all cases, subsequently epigenetic silencing of VMAT2 was defined as a novel adverse predictor of biochemical recurrence after radical prostatectomy (Sorensen et al., 2009).

miRNA on the other hand is a group of small non-coding RNA able to control post-transcriptional gene expression by fine-tuning protein production, through sequence-specific binding within the 3′ untranslated region of mRNA transcripts (Jimenez-Mateos and Henshall, 2013). This translational control of miRNA is observed in neurons (Kosik, 2006), astroytes (Tao et al., 2011) and microglia (Ponomarev et al., 2011) and seems to be involved in the process of epileptogenesis as well as the maintenance and progression of the epileptic state (for review see Jimenez-Mateos and Henshall, 2013). By use of genome-wide miRNA profiling in human hippocampus of autopsy control and mesial TLE patients with and without hippocampal sclerosis, three distinct miRNA signatures were observed. Moreover, deregulated miRNA targets components of key pathways in TLE, including VGLUT1 (Kan et al., 2012). Although several approaches have been examined to modulate individual miRNA expression, such as viral vectors, miRNA decoys and miRNA “sponges,” a better understanding of miRNA biology and function is necessary before an eventually clinical translation of these possible new therapeutic interventions (for review see Brown and Naldini, 2009).

## Conclusion

Epilepsy remains a hard-to-treat disorder for millions of patients. There is an urgent need of non-invasive antiepileptogenic or disease-modifying treatments. VNTs are appealing new intracellular targets for future AEDs. Nevertheless, LEV and brivaracetam are, respectively, hitherto the only approved AED and the only AED currently in the pipeline targeting a vesicular protein (Kaminski et al., 2012). Much more research is needed to improve our understanding of the role of VNTs in normal and pathological conditions, as this will certainly lead to new therapeutic strategies for TLE and other CNS disorders.

### Conflict of interest statement

The authors declare that the research was conducted in the absence of any commercial or financial relationships that could be construed as a potential conflict of interest.
